# Supplementary material for: Photon number resolution without optical mode multiplication
Source: Nanophotonics. 2023 Jan 9;12(3):505–19. doi: 10.1515/nanoph-2022-0614 (PMC11501970; doi:10.1515/nanoph-2022-0614)
Supplement: Supplementary file 1 — Supplementary Material Details [file j_nanoph-2022-0614_suppl.pdf]

Supplementary material for

Photon number resolution without optical mode multiplication

Anton N. Vetlugin,[1,2*] Filippo Martinelli,[1,2] Shuyu Dong,[1,3] and Cesare Soci[1,2,3*]

[1]Centre for Disruptive Photonic Technologies, TPI, Nanyang Technological University, Singapore 637371

[2]Division of Physics and Applied Physics, School of Physical and Mathematical Sciences, Nanyang Technological University, Singapore 637371

[3]School of Electrical and Electronic Engineering, Nanyang Technological University, Singapore 639798

The supplementary material contains three notes: in Note A, the optical response of a single-layer superconducting nanowire single-photon detector (SNSPD) is evaluated for different materials; in Note B, it is shown that quantum state of input light and light source noise plays no role in the efficiency of light absorption; and in Note c, the generalized requirements for a distributed absorber are derived.

A. Optimization of single-layer SNSPD for different materials

In this section, we study the influence of the material on the optical response of a single-layer SNSPD, following Fig. 3 of the main text. We consider conventional superconducting materials: niobium-titanium nitride (NbTiN), niobium nitride (NbN), and molybdenum silicide (MoSi), with the permittivity taken from Ref. [54] in the main text. Since the analytical calculations are based on the transfer matrix method (TMM) with the effective index approximation, we back up our calculations with the finite element method (FEM) simulations performed in COMSOL Multiphysics software.

The optical response of a single-layer SNSPD made of NbTiN is shown in Fig. S1. The unit cell of the FEM model is schematically depicted in Fig. S1A: a 2D model of the nanowire embedded in a matrix of SiO_2 substrate and surrounded by air on top and bottom with periodic conditions on the left and right sides of the unit cell. We note that the material of the surrounding matrix (SiO2 in Fig. S1A) plays a minor role as the effective permittivity of the nanowire layer is mostly defined by the optical constants of the nanowire itself. Polarization is out-of-plane – parallel to the nanowire. The optical response of the nanowire as a function of the nanowire thickness D is shown in Fig. S1B, where analytical calculations (TMM, solid lines) show a good agreement with the simulations (FEM, points). A small difference between $R_{\rm tr}$ (TMM) and $R_{\rm tr}$ (FEM) for thickness $D > D_{\rm opt}$ reflects the limitation of the effective media approximation. This region, $D > D_{\rm opt}$, is out of interest for discussion in the main text. The corresponding circular diagram is shown in Fig. S1C, where the required condition of $t \approx 0.5$ and $r \approx -0.5$ is achieved for the optimal thickness of 30.3 nm (30.6 nm if SiO_2 is replaced by air). The broadband operation of the nanowire (for a fixed optimal thickness) is confirmed in Fig. S1D both analytically (TMM, solid lines) and numerically (FEM, points). The good agreement between the TMM and FEM in the IR region (region of interest) validates the effective index approximation for the nanowire layer. For shorter wavelengths, the approximation breaks down as the slit width (distance between the adjacent nanowires) becomes comparable to the optical wavelength.

Single-layer SNSPDs fabricated out of NbN (Fig. S2) and MoSi (Fig. S3) show similar optical responses where the optimal thicknesses are 32.4 nm (NbN) and 19.7 nm (MoSi). Results presented in Figs. S2 and S3 are close to those in Fig. S1, indicating that methods developed in the main text can be applied to NbN and MoSi SNSPDs.

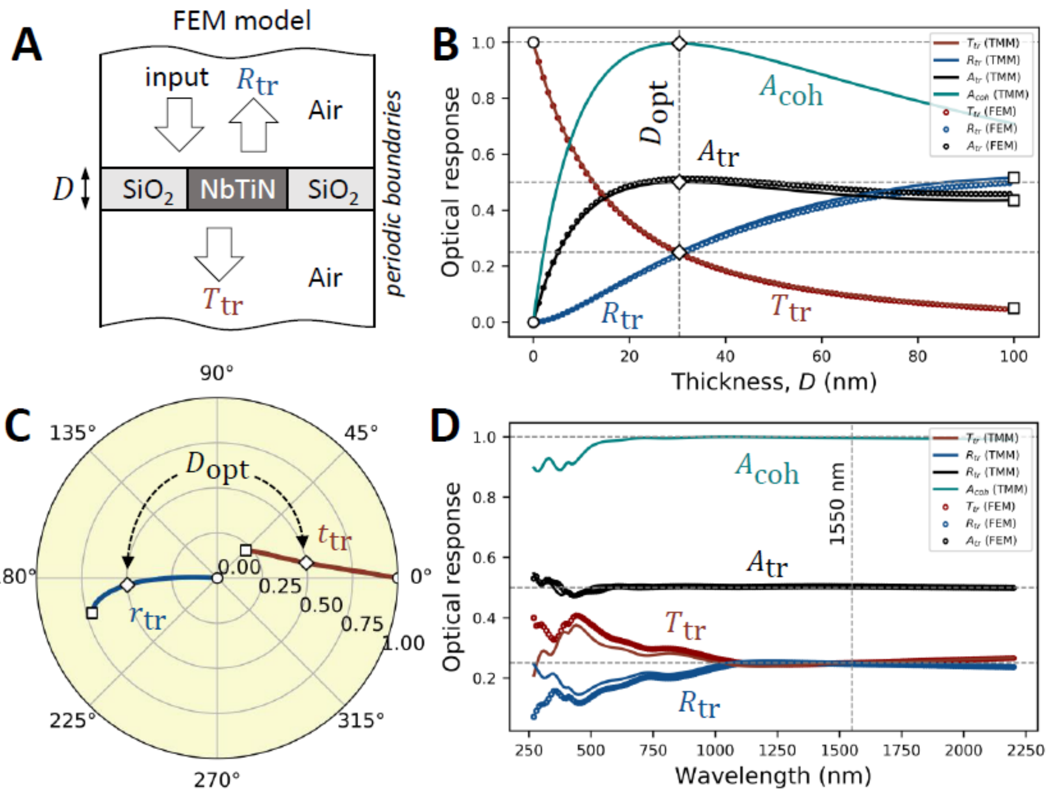

Fig. S1. Optical response of a single layer NbTiN nanowire. A: The nanowire model for numerical FEM calculations. For analytical TMM calculations, the SiO_2-NbTiN-SiO_2 layer of thickness D is approximated by the layer with effective permittivity and of the same thickness. B: Optical response as a function of the layer thickness. C: Circular diagram of the layer. D: Optical spectra of the layer with the optimal thickness D_{opt}.

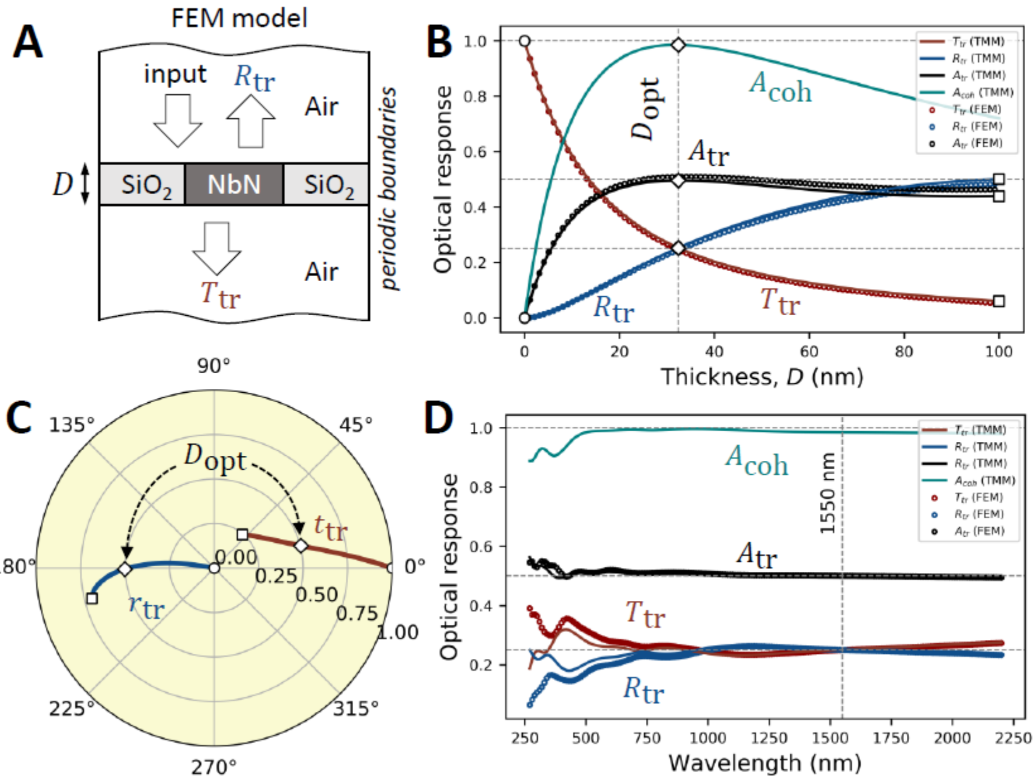

Fig. S2. Optical response of a single layer NbN nanowire.

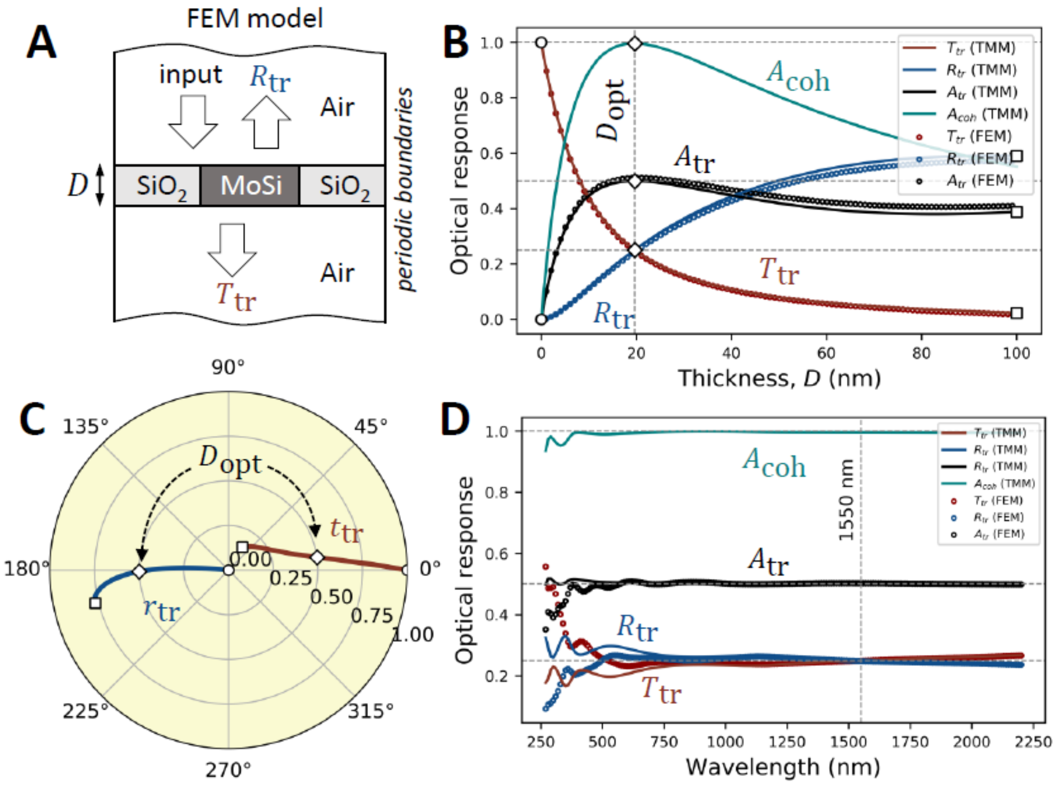

Fig. S3. Optical response of a single layer MoSi nanowire.

B. State-independent absorption of quantum light by coherent detector

The absorption of light in the interferometric detection scheme, shown in Fig. 1A of the main text, is insensitive to the light source noise and quantum state of light. To show this, we consider the transformation of light from the input of the interferometer to its absorption by the detector. The input light with the bosonic amplitude $\hat{a}_0$enters one of the input ports of the 50:50 beamsplitter, Fig. S4. The amplitude of light at the second input port is $\hat{b}_0$, and it is assumed to be in the vacuum state. The output ports of the beamsplitter feed the clockwise ($\hat{a}_1$) and counterclockwise ($\hat{b}_1$) propagating beams of the interferometer:

$$\hat{a}_1 = \frac{1}{\sqrt{2}}(\hat{a}_0 + i\hat{b}_0),$$

$$\hat{b}_1 = \frac{1}{\sqrt{2}}(i\hat{a}_0 + \hat{b}_0).$$

Here amplitude transmission and reflection coefficients of the beamsplitter are $1/\sqrt{2}$ and $i/\sqrt{2}$, respectively. The propagation through the interferometer is associated with the acquisition of the mutual phase difference φ: $\hat{a}_1 \to \hat{a}_1$ and $\hat{b}_1 \to -ie^{i\varphi}\hat{b}_1$, where an additional $-\pi/2$-phase shift was introduced for $\hat{b}_1$ to compensate the phase shift in reflection on the beamsplitter. The detector is a four-port device with two input amplitudes $\hat{a}_1$ and $-ie^{i\varphi}\hat{b}_1$ and two output amplitudes $\hat{a}_2$ and $\hat{b}_2$. Light transformation on the detector is defined by its amplitude transmission (t) and reflection (r) coefficients:

$$\hat{a}_2 = t\hat{a}_1 + re^{i\varphi}\hat{b}_1 + \hat{f}_a,$$

$$\hat{b}_2 = r\hat{a}_1 + te^{i\varphi}\hat{b}_1 + \hat{f}_b.$$

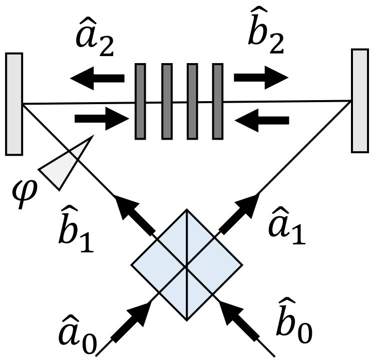

Fig. S4. Transformation of light amplitudes in the interferometric detection scheme.

Here $\hat{f}_a$ nd $\hat{f}_b$ are Langevin noise operators due to the losses of the detector. The absorption of the detector, defined as the ratio between the average intensity of the absorbed and input light,

$$\frac{\langle \hat{a}_0^\dagger \hat{a}_0 \rangle - \langle \hat{a}_2^\dagger \hat{a}_2 + \hat{b}_2^\dagger \hat{b}_2 \rangle}{\langle \hat{a}_0^\dagger \hat{a}_0 \rangle},$$

reaches unity (full absorption) for $t = -r = 1/2$ and $\varphi = \pi$ (or $t = r = -1/2$ and $\varphi = 0$) independent on the state of the input light. In other words, any phase- and state-fluctuations at the input of the interferometer are irrelevant as they equally affect interfering on the detector beams, and only the mutual phase of these two beams defines the absorption level.

Similarly, the absorption of the phase-insensitive detector (Fig. 1B of the manuscript) is independent on the quantum states of light and light source noise. This detector is a two-port device transforming input amplitude $\hat{a}_0$ into output amplitude $\hat{a}_1$:

$$\hat{a}_1 = r\hat{a}_0 + \hat{f},$$

where r is the reflection coefficient of the entire structure, and $\hat{f}$ is the Langevin noise. For $r = 0$, $\langle \hat{a}_1^\dagger \hat{a}_1 \rangle$ equals zero independent of the quantum state of the input light.

C. Derivation of generalized requirements for distributed absorbers

Here we formulate the required optical response of the constituent sublayers for assembling a distributed absorber (detector). This approach is independent of the nature of the constituent sublayers.

Distributed absorber in counter-propagating geometry (phase-sensitive)
In counter-propagating geometry, shown in Fig. 1A of the main text, the distributed detector is illuminated by two coherent beams. Generally, a distributed absorber is composed of *M* identical (to ensure uniform absorption) constituent detectors, each of which is characterized by amplitude transmission (t) and reflection (r) coefficients, and energy absorption coefficients $A = 1 - |t|^2 - |r|^2$. For any thin absorber, t and r are not independent: $t = 1 + r$ (Refs. [42,57] in the main text). All neighboring layers are supposed to be separated by dielectric $\lambda/2$-spacers (absentee layers). First, by "building" the structure layer by layer and calculating the optical response after adding each absorbing layer, we get the recurrent formulas:

$$\tau_1 = t, \qquad \rho_1 = r = t - 1,$$

$$\tau_k = -\frac{\tau_{k-1}}{1-(-1)^k \frac{r}{t}\tau_{k-1}}, \qquad \rho_k = (-1)^{k-1}\tau_k - 1. \tag{S1}$$

Here τ_k and ρ_k are amplitude transmission and reflection coefficients for the structure composed of *k* absorbing layers (with the optical response of t and r each) and *k-1* spacers. For instance, τ_3 is the amplitude transmission coefficient of a structure assembled of three absorbing layers and two

spacers. The alternating sign in (SS1) is due to the change in the transmission phase by π for each added spacer where the total transmission phase of the distributed detector is $(k-1)\pi$. Reflection does not change a sign as each spacer adds a 2π-phase shift to reflection. Due to the absentee spacers, the relation between t and r is translated into a similar relation between τ_k and ρ_k, and it holds until the total thickness of the layers is smaller than the optical wavelength. Transmission of the entire structure,

$$\tau \equiv \tau_M = -\frac{\tau_{M-1}}{1-(-1)^M \frac{r}{t}\tau_{M-1}},$$

can be, by consecutive substitution of (SS1), rewritten as

$$\tau = (-1)^q \frac{\tau_{M-q}}{1-(-1)^{M-q+1} k \frac{r}{t}\tau_{M-q}},$$

for $1 \leq q \leq M-1$. Finally, going down to the $q = M-1$, τ is expressed as a function of t and *M:*

$$\tau = (-1)^{M-1} \frac{t}{M-(M-1)t}. \quad \text{(S2)}$$

Claiming $\tau = 1/2$ (for even *M*) or $\tau = -1/2$ (for odd *M*), the optical response of the constituent detectors is found to be

$$t = \frac{M}{M+1}, \qquad r = -\frac{1}{M+1}, \qquad \text{and } A = \frac{2M}{(M+1)^2}. \quad \text{(S3)}$$

for a distributed absorber with *M* layers, each constituent absorbing layer should satisfy (SS3).

Distributed absorber in the Salisbury screen geometry (phase-insensitive)

In the Salisbury screen geometry, shown in Fig. 1B of the main text, the distributed absorber (detector) is placed on top of the reflector with a dielectric $\lambda/4$-spacer in between. In a sense, the reflector mirrors the entire space on the left side, including input light and constituent detectors, so that, effectively, the counter-propagating in-phase geometry is reproduced, and the number of detectors is doubled. In this case, the reflection coefficient ρ_S of the entire device is

$$\rho_S = \rho' + \frac{{\tau'}^2}{1-\rho'},$$

where ρ' and τ' are the reflection and transmission coefficients of the distributed detector with K layers. It is assumed that the mirror is perfect with unity reflectivity. We are looking for optical parameters of each layer: t', $r' = t' - 1$, and A'. Similarly to (SS1), $\rho' = (-1)^{K-1}\tau' - 1$, and, by imposing condition of zero total reflection, $\rho_S = 0$, we get the optical response of the distributed detector:

$$\tau' = \pm\frac{2}{3},$$

where "+" and "-" hold for odd and even K, respectively. Substituting τ' instead of τ into (SS2), we arrive to:

$$t' = \frac{2K}{2K+1}, \qquad r' = -\frac{1}{2K+1}, \qquad \text{and } A' = \frac{4K}{(2K+1)^2}. \quad \text{(S4)}$$
